# Supplementary material for: For clinical data extraction, QLoRA attains accuracy close to LoRA while requiring lower compute resources
Source: medRxiv. 2025 Oct 23:2025.10.21.25338506. Preprint. [Version 1] doi: 10.1101/2025.10.21.25338506 (PMC12633606; doi:10.1101/2025.10.21.25338506)
Supplement: Supplement 1 [file NIHPP2025.10.21.25338506v1-supplement-1.pdf]

medRxiv preprint doi: https://doi.org/10.1101/2025.10.21.25338506; this version posted October 23, 2025. The copyright holder for this preprint
(which was not certified by peer review) is the author/funder, who has granted medRxiv a license to display the preprint in perpetuity.
It is made available under a CC-BY 4.0 International license .

Annex 1: Figures and Tables

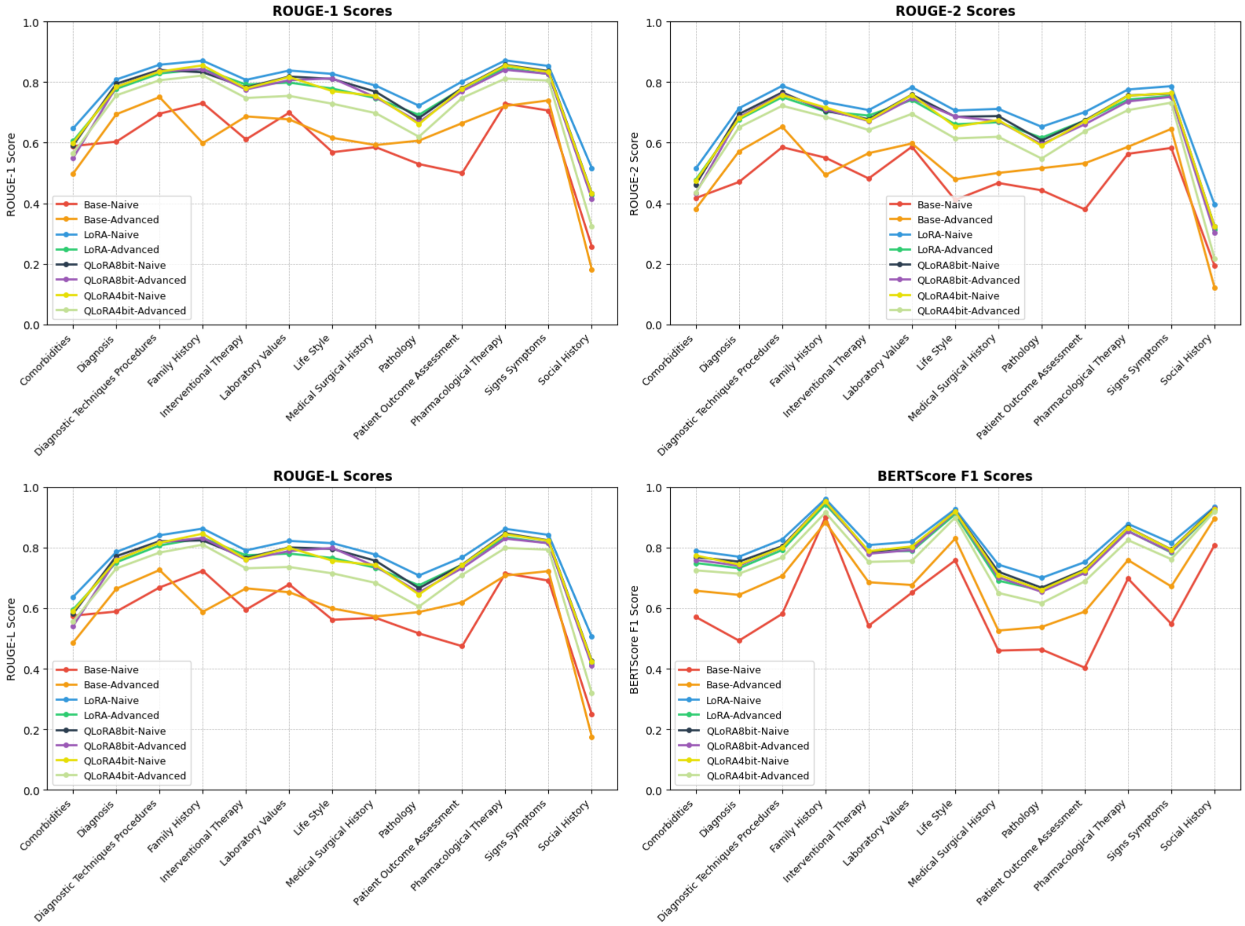

medRxiv preprint doi: https://doi.org/10.1101/2025.10.21.25338506; this version posted October 23, 2025. The copyright holder for this preprint
(which was not certified by peer review) is the author/funder, who has granted medRxiv a license to display the preprint in perpetuity.
It is made available under a CC-BY 4.0 International license .

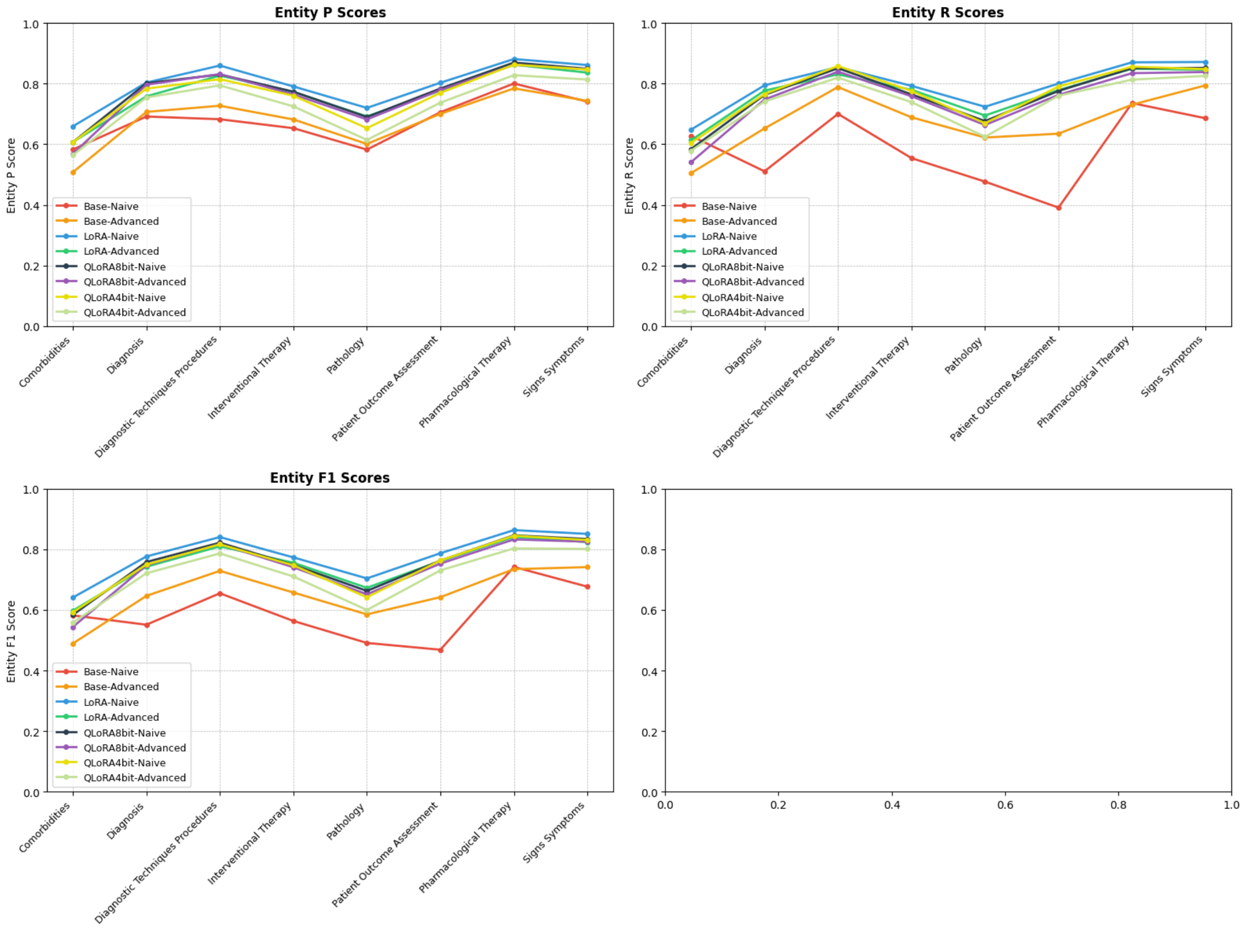

Figure S1 : Model Performance across structured data extraction categories (ROUGE, BERT and Entity level Precession(P), Recall(R) and F1 score).

Table S1: Comparison of naïve prompt results of 4bit-QLoRA fine-tuned model trained for 1 epoch vs 3 epoch.							
Model	B-F1	R1	R2	RL	E-F1	E-P	E-R
QLoRA4bit 3epoch	0.82	0.79	0.69	0.77	0.78	0.79	0.79
QLoRA4bit 1epoch	0.80	0.75	0.65	0.73	0.79	0.76	0.77

medRxiv preprint doi: https://doi.org/10.1101/2025.10.21.25338506; this version posted October 23, 2025. The copyright holder for this preprint (which was not certified by peer review) is the author/funder, who has granted medRxiv a license to display the preprint in perpetuity.
It is made available under a CC-BY 4.0 International license .

Annex 2: Prompts

Naïve Prompt

You are a helpful assistant. I will provide you a clinical report and I want you to extract several fields from it as strings and not lists.
If a field is not mentioned on the report provide the value "N/A", otherwise provide the extracted text.

YOU MUST ONLY OUTPUT THE JSON!
NO OTHER TEXT OR EXPLANATION.

JSON fields:

```
{
    "life_style": "",
    "family_history": "",
    "social_history": "",
    "medical_surgical_history": "",
    "signs_symptoms": "",
    "comorbidities": "",
    "diagnostic_techniques_procedures": "",
    "diagnosis": "",
    "laboratory_values": "",
    "pathology": "",
    "pharmacological_therapy": "",
    "interventional_therapy": "",
    "patient_outcome_assessment": "",
    "age": "",
    "gender": ""
}
```

medRxiv preprint doi: https://doi.org/10.1101/2025.10.21.25338506; this version posted October 23, 2025. The copyright holder for this preprint (which was not certified by peer review) is the author/funder, who has granted medRxiv a license to display the preprint in perpetuity.
It is made available under a CC-BY 4.0 International license .

Advanced Prompt

TASK DESCRIPTION:

You are given a clinical report. Your task is to extract key information from the unstructured text and convert it into a structured JSON format. Each field should be populated with relevant details from the report, and if multiple pieces of information exist for a field, they should be separated by semicolons. If a particular field's information is not available in the report, use "N/A" (Not Available) for that field. Use the following structure for the output:

Field Descriptions:

- **Life Style**: Extract information related to the patient's habits and daily activities, such as smoking status, alcohol consumption, exercise, diet, occupation, and living environment.
- **Family History**: Extract any relevant medical conditions or genetic disorders that run in the patient's family, such as a history of heart disease, diabetes, or cancer.
- **Social History**: Extract details about the patient's social background, including marital status, support systems, substance use, and housing situation.
- **Medical/Surgical History**: Extract any past medical conditions, chronic diseases, previous surgeries, hospitalizations, or treatments the patient has undergone.
- **Signs and Symptoms**: Extract the patient's current symptoms, their duration, and severity.
- **Comorbidities**: Extract any other medical conditions the patient has that coexist with the primary diagnosis, particularly those that may impact treatment.
- **Diagnostic Techniques and Procedures**: Extract details of any diagnostic tests, imaging, or procedures performed, such as X-rays, MRIs, blood tests, or biopsies.
- **Diagnosis**: Extract the primary diagnosis as well as any secondary diagnoses or differential diagnoses.
- **Laboratory Values**: Extract specific laboratory test results, including values like blood counts, electrolyte levels, or any other relevant lab data.
- **Pathology**: Extract findings from any pathological examinations, including biopsy results, histopathology, or cytology.
- **Pharmacological Therapy**: Extract details of any medications prescribed, including names, dosages, frequency, and duration of treatment.
- **Interventional Therapy**: Extract information on any surgical or non-surgical interventions, such as procedures performed and their outcomes.
- **Patient Outcome Assessment**: Extract the assessment of the patient's current health status, functional status, quality of life, and any follow-up plans.
- **Age**: Extract the patient's age at the time of the report.
- **Gender**: Extract the patient's gender as stated in the report.

medRxiv preprint doi: https://doi.org/10.1101/2025.10.21.25338506; this version posted October 23, 2025. The copyright holder for this preprint (which was not certified by peer review) is the author/funder, who has granted medRxiv a license to display the preprint in perpetuity.
It is made available under a CC-BY 4.0 International license .

Important Note:

- If any information is not available in the clinical report, use "N/A" (Not Available) for that field.

Instructions:

- Populate each field based on the information in the clinical report.
- Use semicolons to separate multiple entries within a single field.
- Use "N/A" (Not Available) for fields where information is not provided in the report.
- Ensure accuracy and completeness for each category.

YOU MUST ONLY OUTPUT THE JSON!

NO OTHER TEXT OR EXPLANATION.
